# Colitis-associated intestinal microbiota regulates brain glycine and host behavior in mice

**DOI:** 10.1038/s41598-022-19219-z

**Published:** 2022-09-29

**Authors:** Maryana V. Morozova, Mariya A. Borisova, Olga A. Snytnikova, Kseniya M. Achasova, Ekaterina A. Litvinova, Yuri P. Tsentalovich, Elena N. Kozhevnikova

**Affiliations:** 1 Scientific Research Institute of Neurosciences and Medicine (SRINM), Novosibirsk, 630117 Russian Federation; 2 Institute of Molecular and Cellular Biology SB RAS, Novosibirsk, Russian Federation; 3 The Federal Research Center Institute of Cytology and Genetics SB RAS, Novosibirsk, Russian Federation; 4 International Tomography Center SB RAS, Novosibirsk, Russian Federation; 5 Center of Technological Excellence, Novosibirsk State Technical University, Novosibirsk, Russian Federation; 6 Novosibirsk State Agrarian University, Novosibirsk, Russian Federation

**Keywords:** Microbiology, Molecular biology, Neuroscience

## Abstract

Inflammatory bowel diseases (IBD) are chronic and relapsing inflammatory disorders of the gastrointestinal tract with complex etiology and no strategies for complete cure. IBD are often complicated by mental disorders like anxiety and depression, indicating substantial shifts in the microbiota gut-brain axis. However, the mechanisms connecting IBD to mental diseases are still under debate. Here we use *Muc2* knockout mouse model of chronic colitis to uncouple the effects of the intestinal microbiota on host behavior from chronic inflammation in the gut. *Muc2* knockout male mice exhibit high exploratory activity, reduced anxiety-related behaviors, impaired sensorimotor gating, and altered social preference towards males and females. Microbial transfer to wild-type mice via littermate co-housing shows that colitis-associated microbiota rather than inflammation per se defines behavioral features in *Muc2* colitis model. Metagenomic profiling and combination of antibiotic treatments revealed that bacterial species *Akkermansia muciniphila* is associated with the behavioral phenotype in mutants, and that its intestinal abundance correlates with social preference towards males. Metabolomic analysis together with pharmacological inhibition of Gly and NMDA receptors helped us to determine that brain glycine is responsible for the behavioral phenotype in *Muc2* mice. Blood and brain metabolic profiles suggest that microbiota-dependent changes in choline metabolism might be involved in regulation of central glycine neurotransmission. Taken together, our data demonstrates that colitis-associated microbiota controls anxiety, sensorimotor gating and social behavior via metabolic regulation of the brain glycinergic system, providing new venues to combat neurological complications of IBD.

## Introduction

IBD are gastrointestinal (GI) disorders involving inflammation, ulcerations, epithelial barrier dysfunction, diarrhea, weight loss, anemia and predisposition to colorectal cancer^1–4^. IBD onset is on average at 15–35 years of age, and it remains a chronic and relapsing disorder throughout patient’s life. The etiology of IBD is under debate, with genetic predisposition, environmental exposures and diet emerging as the main risk factors^5–7^. Human studies support the role of heritable aspects in IBD, since first-degree relatives of IBD patients have significantly higher risk to develop this illness than the background population^8–10^. Genome-wide screens revealed about 200 genetic loci linked to IBD, many of which are immune system components^11–13^. Animal models demonstrate that mutations in some of these genes predispose to intestinal inflammation upon environmental triggers, but may not be sufficient to induce colitis^14^. These data suggest that IBD arise on the interface of mucosal and intestinal environment, both being indispensable for the disease progression^15^. The main component of the heritable intestinal environment is microbiota, which is in a mutual regulatory relationship with mucosal immune system^16–18^. Indeed, multiple reports describe a significant association of intestinal inflammation with distinct microbiome signatures that might induce IBD as well as result from it^19–21^. In the experimental models of colitis, transplants of fecal microbiota or inoculation with probiotic bacterial species alleviated the severity of inflammation^22–24^. Therefore, intestinal microbes can beneficially regulate pathological aspects of IBD.

It has been noticed that IBD are often accompanied by mental disorders including anxiety, depression, bipolar disorder, insomnia, lack of motivation, and others^25,26^. About 35–40% of IBD patients suffer from anxiety and about 22–25%—from depression, indicating a strong interaction between intestinal inflammation and the central nervous system (CNS)^25,27,28^. Particularly, microbial metabolism substantially impacts the overall metabolomic profile of the host, including amino acids, short chain fatty acids, lipids and intermediary metabolites^29–31^. These microbiota-associated molecules can potentially affect host neurophysiology and behavior serving as precursors for neurotransmitter biosynthesis and by regulating neuro- and axonogenesis^32–35^. However, the mechanisms underlying mental disorders during IBD are still unclear due to the complexity of this disease. In order to understand the interrelation between chronic colitis, microbiota and CNS function, we utilized *Mucin 2* (*Muc2*) knockout mice as a well-described model of intestinal inflammation^36,37^. This mutant animals lack the major secreted mucin in the intestine and reconstitute key features of chronic colitis^38–40^. Here we show that intestinal microbiota regulates brain glycine and controls anxiety-related and social behaviors in *Muc2* knockout mice. These behavioral traits were associated with elevated intestinal *Akkermansia muciniphila* and were transferrable along with the intestinal microbiota to wild-type animals. We propose a mechanism, by which *Akkermansia muciniphila* affects host betaine-glycine metabolism leading to activation of glycine-dependent neurotransmission. Therefore, glycine emerges as a potential mediator of the microbiota gut-brain axis regulated by intestinal microbiota.

## Materials and methods

### Resource availability

#### Lead contact

Further information and requests for laboratory resources and reagents should be directed to and will be fulfilled by the corresponding author, Elena N. Kozhevnikova (kozhevnikovaen@physiol.ru).

### Materials availability

This study did not generate new unique reagents.

### Experimental model and subject details

All reagents and resources used in this study are given in the Resources table.

**Table:** 

Reagent or resource	Source	Identifier
**Chemical**s		
Methanol (HPLC grade)	Scharlau	Cat # ME03152500
Chloroform for liquid chromatography	Merck	Cat #: 67-66-3
D2O (99.9%)	Cambridge Isotope Laboratories Inc	Code: DLM-4-100
DSS (sodium 4,4-dimethyl-4-silapentane-1-sulfonate)	Cambridge Isotope Laboratories Inc	Code: DLM-32-1
Vancomycin	Krasfarma	ATC code J01XA01
Rifampicin	Belmedpreparaty	ATC code J04AB02
Amoxicillin + clavulanic acid	Sandoz Lek d.d., Ljubljana	ATC code J01CR02
Strychnine	Sigma‐Aldrich	Cat # S0532
L‐701,324 (7‐chloro‐4‐hydroxy‐3(3‐phenoxy)phenylquinoline‐2‐(H)‐one)	Sigma‐Aldrich	Cat # L0258
DMSO	Amresco	Cat # Am-O231-0.1
Hematoxylin and Eosin Staining Kit	Abcam	Cat # ab245880
Blue food coloring	KREDA	N/A
TRIzol reagent	Thermo Fisher Scientific	Cat # 15596026
M-MuLV reverse transcriptase	SibEnzyme	Cat # E317
**Critical commercial assays**		
QIAamp DNA Stool Mini Kit	Qiagen	Cat # 51504
BioMaster HS-qPCR SYBR Blue	BioLabMix	Cat #MHC030-400
Phusion® High-Fidelity PCR Master Mix	New England Biolabs	Cat #M0531S
Qiagen Gel Extraction Kit	Qiagen	Cat #28706X4
NEBNext® UltraTM DNA Library Prep Kit for Illumina sequencing	New England Biolabs	Cat #E7370L
**Deposited data**		
https://data.mendeley.com/datasets/y85tx8hzdh/draft?a=5fde3ad3-1078-4911-9523-92a361805999	Mendeley	Metagenomic analysis of 16S rRNA amplicons
amdb.online/amdb/experiments/152/ amdb.online/amdb/experiments/154/	Animal Metabolite Database	Blood metabolomics Brain metabolomics
**Experimental models: Organisms/strains**		
C57BL/6JNskrc	ICG SB RAS	C57BL/6J
Muc2tm1Avel on C57BL/6JNskrc genetic backgound	ICG SB RAS	C57BL/6 103
BALB/c	ICG SB RAS	BALB/c J
**Oligonucleotides**		
See Table S1		
**Software and algorithms**		
Ethovision XT 10 software	Noldus	https://www.noldus.com/ethovision-xt
STATISTICA12	StstSoft	http://statsoft.ru/
**Other**		
Individually ventilated cages	Optimice, Animal Care Systems	https://animalcaresystems.com/optimice-system/
Food	SSniff	Cat #V1185-300
Open field test installation	San Diego Instruments	https://sandiegoinstruments.com/product/pas-open-field/
Light–dark test installation	N/A	N/A
Elevated plus maze installation	San Diego Instruments	https://sandiegoinstruments.com/product/elevated-plus-maze/
Glass marbles, d = 1.0 cm	N/A	N/A
Tail Suspension Test Cubicle	N/A	N/A
Forced Swim Test Cylinder for Mouse	Med Associates, Inc	ENV-590 M
PhenoMaster device	TSE Systems	https://www.tse-systems.com/service/phenomaster/
Rotarod device	Neurobotics	https://neurobotics.ru/en/catalog/animal-physiology-en/rotarod-en/
T-maze test installation	Med Associates, Inc	MED-TMMN-AD
Barnes maze	Med Associates, Inc	ENV-562-M
IntelliCage setting	TSE Systems	https://www.tse-systems.com/service/intellicage/
Gemini Avoidance System apparatus	San Diego Instruments	https://sandiegoinstruments.com/product/gemini-active-and-passive/
SR-Pilot device	San Diego Instruments	https://sandiegoinstruments.com/product/sr-lab-startle-response/
Social preference test installation	N/A	N/A
NMR spectrometer	Bruker BioSpin	AVANCE III HD 700 MHz

#### Animals

The experiments were performed in the Center for Genetic Resources of Laboratory Animals at the Federal research center Institute of Cytology and Genetics of The Siberian Branch of the Russian Academy of Sciences (ICG SB RAS) and in the Scientific Research Institute of Neurosciences and Medicine (SRINM). All procedures were conducted under Russian legislation according to the standards of Good Laboratory Practice (directive # 267 from 19.06.2003 of the Ministry of Health of the Russian Federation), institutional bioethical committee guidelines and the European Convention for the protection of vertebrate animals. All procedures were approved by the Bioethical committee at SRINM, protocol #8 dated 15.08.2019. All animals had SPF status, which was tested quarterly according to Federation of European laboratory animal science association’s (FELASA) recommendations^73^. All results are reported in a manner consistent with ARRIVE guidelines^74^.

**Figure 1:**
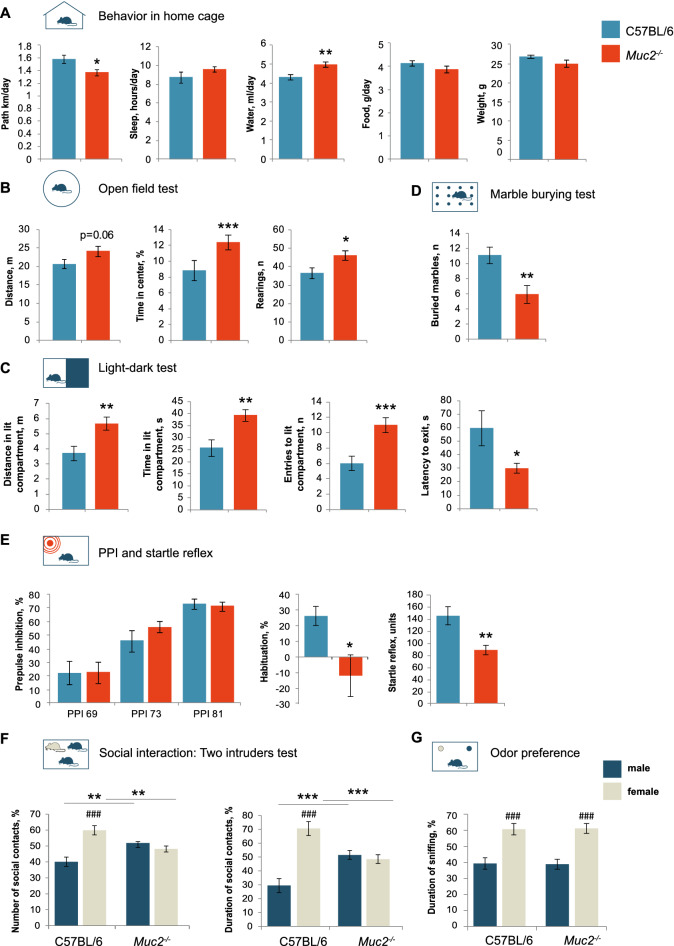
Behavioral traits of *Muc2*^*−/−*^ animals. (**A**) Behavior in home cage (*n* = 9–12). Motor activity: *t* = 2.46, *p* = 0.024; water consumption rate: *t* = − 3.59, *p* = 0.002, Student's *t*-test. (**B**) Open field test (*n* = 18–20). Distance: *t* = − 1.91, *p* = 0.06, Student's *t*-test; rearings: *t* = − 2.326, *p* = 0.026, Student's *t*-test; time in the center: Z = − 3.333, *p* < 0.001, Mann–Whitney *u* test *vs*. C57BL/6. (**C**) Light–dark test (*n* = 17–20). Distance: *t* = − 3.067, *p* = 0.004; time: *t* = − 3.08, *p* = 0.004; entries: *t* = − 3.66, *p* < 0.001, Student's *t*-test. (**D**) Marble burying test (*n* = 17–20). Number of buried marbles: Z = 2.93, *p* = 0.003; Mann–Whitney *u* test. (**E**) Habituation and startle reflex (n = 16–18). Startle reflex: *t* = 3.246, *p* = 0.003, Student's *t*-test; habituation: Z = 2.17, *p* = 0.03; Mann–Whitney *u* test. (**F**) Two intruders test (*n* = 10–11). There was a statistically significant interaction between the resident genotype and the intruder gender (number of contacts: F(1, 38) = 21.990, *p* < 0.001; duration: F(1, 38) = 26.045, *p* < 0.001, two-way ANOVA). Number of contacts (C57BL/6, male *vs.* female): *p* < 0.001, Fisher’s LSD test; duration (C57BL/6, male *vs.* female): *p* < 0.001, Fisher’s LSD test. Number of contacts with a male/female intruder *vs*. C57BL/6: *p* < 0.01, Fisher’s LSD test; duration of contact with a male/female intruder *vs*. C57BL/6: *p* < 0.001, Fisher’s LSD test. (**G**) Odor preference test (*n* = 12–21). There was a statistically significant influence of the gender of animal whose odor was presented to the tested male F(1, 62) = 34.17 *p* < 0.001 two-way ANOVA. Duration of sniffing (male *vs.* female): *p* < 0.001, Fisher’s LSD test. * = *p* < 0.05, ** = *p* < 0.01, *** = *p* < 0.001, *vs*. C57BL/6. ### = *p* < 0.001, male *vs*. female.

**Figure 2:**
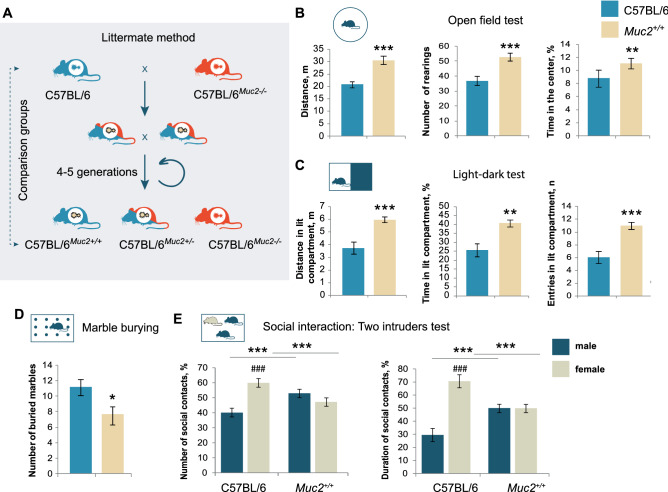
Behavioral traits are associated with microbiota of *Muc2*^*−/−*^ mice. (**A**) The scheme of the littermate co-housing method. (**B**) Open field test (*n* = 18–20). Distance: *t* = − 3.895, *p* < 0.001; rearings: *t* = − 4.97, *p* < 0.001, Student's *t*-test; time in the center: Z = − 2.997, *p* < 0.001, Mann–Whitney *u* test. (**C**) Light–dark test (*n* = 17–20). Distance: *t* = − 4.019, *p* < 0.001; time: *t* = − 3.584, *p* < 0.001; entries: *t* = − 4.26, *p* < 0.001, Student's *t*-test. (**D**) Marble burying test (*n* = 18–20). Number of buried marbles: Z = 2.22, *p* = 0.026; Mann–Whitney *u* test. (**E**) Two intruders test (*n* = 10–11). Two-way ANOVA revealed a statistically significant interaction between the resident group and the intruder gender (number of contacts: F(1, 38) = 15.490, *p* < 0.001; duration: F(1, 38) = 28.306, *p* < 0.001 Number of contacts (C57BL/6, male *vs.* female): *p* < 0.001, Fisher’s LSD test; duration (C57BL/6, male *vs.* female): *p* < 0.001, Fisher’s LSD test. Number of contacts with a male/female intruder *vs*. C57BL/6: *p* = 0.008, Fisher’s LSD test; duration of contact with a male/female intruder *vs*. C57BL/6: *p* < 0.001, Fisher’s LSD test. * = *p* < 0.05, ** = *p* < 0.01, *** = *p* < 0.001, *vs*. C57BL/6. ### = *p* < 0.001, male *vs*. female.

The study was conducted using C57BL/6JNskrc (our in-house C57BL/6J sub-colony) and *Muc2*^*−/−*^ mouse strains. C57BL/6JNskrc were recovered from C57BL/6J F1 frozen embryos using SPF CD1 females as foster mothers. *Muc2*^*−/−*^ mice were obtained by the rederivation of previously published *Muc2*^*tm1Avel*^*/Muc2*^*tm1Avel*^ mice^75^ with C57BL/6 genetic background^38^ in SPF CD1 female mice and back-crossed to C57BL/6JNskrc. Mutant animals and their wild-type littermates (*Muc2*^+*/*+^ mice) were generated by crossing *Muc2*^+*/−*^ females to *Muc2*^+*/−*^ males. In behavioral phenotyping, C57BL/6JNskrc (C57BL/6 further on) mice were used as a control group (Figs. 1 and 2). Wild type littermates (*Muc2*^+*/*+^ further on) served as a comparison group where indicated. Ten adult male BALB/c mice were used as a live object in the social preference test, 10 adult male and 10 adult estrus females were used in social tests. The female's fur on the back was stained with blue food coloring^76^ to discriminate between males and females in the two intruder test. BALB/c mice were used as intruders for convenience as they were easily discriminated from the resident mice by fur color.

Adult 8–14 week-old male mice were housed in groups of the same-sex siblings in individually ventilated cages in 12 h/12 h light/dark photoperiod under standard conditions. Food and water were provided ad libitum. Some SPF *Muc2* mice develop severe intestinal prolapses and exhibit substantial weight loss after weaning. None of these animals was included into the test groups. Sucrose preference test was performed using 8–14 week-old female mice housed under the same conditions.

### Method details

#### Co-housing method

Littermate co-housing: *Muc2*^*−/−*^ mice were crossed to C57Bl/6 to generate heterozygous mutants (*Muc2*^+*/−*^). *Muc2*^+*/−*^ animals were crossed for five generations to obtain *Muc2*^+*/*+^ and *Muc2*^*−/−*^ littermates used in this study.

Direct co-housing was performed in order to control for potential mutations that could accumulate in *Muc2*^+*/*+^ mice during the five generation of *Muc2*^+*/−*^ crosses. It was performed as follows: *Muc2*^+*/−*^ female mice were crossed to *Muc2*^+*/−*^ males. C57BL/6 females were crossed to C57BL/6 males. Pregnant *Muc2*^+*/−*^ and C57BL/6 were housed in separate open cages next to each other with an exchange of dirty litter twice a week. Upon weaning, *Muc2*^+*/*+^ and C57BL/6 progeny were labelled, mixed and co-housed in same cages until behavioral tests were completed^77^.

#### Behavioral tests

The order of the behavioral tests with males were as follows: home cage activity, open field, light/dark box, elevated plus maze, marble burying test, Barnes maze, social preference, T- maze, rotarod, tail suspension, forced swim test, startle reflex, active avoidance. For each animal, the tests were performed 3 days apart. Sucrose preference was performed on a separate group of females used for this test only. Timeline of behavioral phenotyping is given in Supplementary Table 1.

All behavioral tests were conducted in the dark time. Three days before the experiment, mice were placed into individual cages to avoid the group effects. For each experiment, a test group and a control group were tested on the same day. In some cases, all groups were divided so that at least some test and control animals are tested on the same day in consecutive days, and the results for each group were combined. The mouse behavior during testing was recorded and processed using Ethovision XT 10 software except for a number of behavioral test that were analyzed manually as specified further.

Animals were euthanized using CO_2_ inhalation. Descending colon and brain samples were taken for nuclear magnetic resonance (NMR) metabolomic profiling and gene expression analyses. Intestinal contents were collected for metagenomic and PCR analyses.

#### Home-cage behavior

Home cage behavior was evaluated in order to measure animals’ behavior in a familiar setting without novelty or stressors that usually accompany other behavioral tests. Home cage behavior was studied using PhenoMaster automated home cage phenotyping setup according to the manufacturer’s instructions. Mice were individually housed for three days in PhenoMaster cages. Locomotion, food and water intake and sleep duration were recorded during the second and the third day of housing. A mouse was considered asleep if no movements were detected in 40 s or longer^78^. Animal weight was measured manually.

#### Open field test

Open field test measures motor and exploratory activity, and can also reflect general anxiety in a test animal. A mouse was placed in a center of a square plastic setting sized 40 cm × 40 cm with transparent walls and an opaque bottom under white light. Central square 20 cm × 20 cm in size were considered as the center. Animal’s movements were recorded by an overhead video camera and the rearings were detected by a side-view video camera for 6 min. Total distance, time spent in the center of the arena, the number of rearings and the number of entries in center were analyzed using the Ethovision XT10 software.

#### Light–dark test

Light–dark test measures anxiety, exploratory and motor behavior. The light–dark test apparatus consisted of a box (42 × 21 ×  25 cm ) divided into a smaller (one-third) dark compartment and a large (two-thirds) illuminated compartment. Two chambers were connected via a 3 cm × 4 cm opening. A mouse was placed in the middle of the dark chamber, facing away from the opening. During 5 min of test, the distance, time and entries into the lit compartment as well as latency to escape after the initial placement were recorded using Ethovision XT10 software^79,80^.

#### Elevated plus maze

Elevated plus maze is usually used to evaluate anxiety. A mouse was placed in the center of the maze facing an open arm. The distance in open arms, the number of head dips, the time spent in the open arms and the number of entries in open arms were measured during the 5-min observation period using Ethovision XT 10 software^81^.

#### Marble burying

Marble burying test measures repetitive behavior that can reflect autistic-like traits or anxiety-like behavior. Twenty glass marbles (d =  1.0 cm ) were evenly distributed on top of a 4 cm layer of sawdust in the plastic animal cage ( 37 cm  ×  21 cm  ×  15 cm ). A mouse was placed in the cage and allowed to move freely. After 30 min of exposure, the animal was removed. The number of marbles buried at least two-thirds deep were counted.

#### Tail suspension test

Tail suspension test is employed to evaluate depressive-like behavior. The mouse was hung for 6 min by the tail with a strip of masking tape on a horizontal bar at a height of 30 cm . The mouse began to move, trying to free itself and attempted to climb on the tail or hung motionless. The total immobility time in seconds was recorded with the Ethovision XT 10 software. The number of animals that successfully climbed on the tail was recorded manually.

#### Forced swim test

Forced swim test is another method to measure depressive-like behavior. Animals were placed into a glass cylinder (d = 10 cm, h = 44 cm) filled up to 25 cm with tap water at a temperature of 25 °C. After 2 min of adaptation, the total time of immobility (sec) was recorded with the Ethovision XT 10 software for 4 min^82^. An experimenter constantly monitored the animals during the test.

#### Rotarod test

Rotarod test allows evaluating motor skill. The Rotarod device consisted of a metal frame with a motorized rotating assembly of rods. The mouse was placed on the rod facing the direction of rotation followed by the start of rotation. The rotational speed was increased incrementally by 3 rpm every 10 s. Rotational speed ranged from 0 to a maximum speed of 30 rpm for a maximum period of 2 min. Mice were tested 2 times using the same protocol with an interval of 20 min. The duration of walking before falling and maximal rotational speed before falling were recorded using Rotarod computer software and averaged between the two tests.

#### T-maze spontaneous alternation test

T-maze spontaneous alternation test was used to measure short-term spatial memory and exploration. T-maze was carried out in red light (28 lx) in a T-shaped apparatus with two closed side compartments (right and left arms) and a start arm separated by automatic doors. A mouse was initially placed in the start arm for 5 s, after which the start arm door and one goal arm door were opened allowing the animal to examine only one goal arm. The opening goal arm was random for all mice. The door closed upon entering the goal arm, and after 5 s, the mouse was returned to its home cage. After that, the apparatus was cleaned to eliminate animal odor traces, and the mouse was placed to the start arm again for 5 s. The time between trials was 60 s. Then the start arm door and both goal arm doors were opened so that the animal could examine any goal arm. Once it was selected, the opposite goal arm door closed. After 5 s the mouse was returned to its home cage, the apparatus was cleaned, and the latter session was repeated 14 times for each test animal. Maximal duration of a session was 90 s. If no choice was made, the animal was returned to its home cage^83^. The total alternation rate was expressed as a percentage of the correct arm selection (opposite to the previous choice) of the total number of sessions with free arm choice.

#### Barnes maze

Barnes maze was used to assess spatial learning and memory. The mouse was placed in a open circular 120 cm-wide platform located 90 cm above the floor with 40 circular holes (8 cm in diameter) evenly pierced around the periphery. The escape box was located under one of the holes, one of four preset locations was randomly chosen for each mouse and did not change during the experiment. The test was carried out in bright white light (about 1000 lx) to stimulate the desire to escape. Distant cues were placed on the walls and floor visible from the platform to help animals navigate the process of searching for the escape hole. The test was composed of four stages as follows:

*Habituation* (1 day, 2 trials of 5 min): the mouse was placed close to the target hole, and if after 5 min the animal did not find the escape box, it was carefully guided and left in the escape box for 2 min.

*Training* (4 consecutive days, 2 trials per day for 5 min): the mouse was placed in the center of the platform, and the latent time to find the escape box and the distance traveled to the target hole were recorded using Ethovision XT10 software. In case a mouse failed to find a target hole, it was guided there and left in the escape box for 2 min. The trial was repeated after 30 min.

*Testing* (1 day, 1 trial of 5 min): the mouse was placed in the center of the platform and the latency to escape, the distance traveled to the target hole, time spent near the escape hole, and serial number of the first visited hole counting from the escape hole were recorded using Ethovision XT10 software^84–86^.

*Long-term memory retention* was evaluated on day 12 (1 day, 1 trial of 5 min): the mouse was placed in the center of the platform and the latency to escape and the distance traveled to the target hole were recorded using Ethovision XT10 software. No training was conducted between the 5th and 12th day of the experiment^86^.

#### Sucrose preference test

Sucrose preference was evaluated in the IntelliCage setting. It allowed testing hedonic response, and might help to detect depressive-like traits. The IntelliCage apparatus consists of a polycarbonate cage ( 58 cm  × 40 × 20.5 cm) with two drinking bottles in each corner and equipped with automatic doors and sensors recording entries of an animal, nose pokes, and licking. The cage floor was covered with the sawdust and used as home cage starting the 4^th^ day of the experiment. The female mice were used in order to avoid aggressive behavior after placement of non-sibling adult animals into the setting. Testing was carried out in two apparatus cages: the first cage contained C57BL/6 females, and the second cage—*Muc2*^*−/−*^ females. Each mouse was labelled with a code of an implanted radio-frequency identification chip.

The following protocol was used:

*Chip adaptation*—3 days after chip implantation female mice were kept in their home cages.

*Adaptation to IntelliCage*—7 days. Mice were placed in the IntelliCage supplied with drinking water and food ad libitum. All doors in corners were open and animals had free access to water.

*Sucrose preference*—5 days. Drinking water was replaced with 2% sucrose in 2 corners out of 4, all doors remained open^87,88^. The IntelliCage Software automatically recorded entries and licks. Sucrose preference was calculated as the number of sucrose licks in percent of the total number of licks.

#### Active avoidance test

Active avoidance test was used to evaluate learning and memory. The Gemini Avoidance System apparatus consisted of two automated shuttle-boxes, each one was divided into two 20 ×  10 cm compartments, connected with a 3 ×  3 cm opening. At the beginning of each test day, an animal was given a 5-min adaptation period in the apparatus and was allowed to move freely. The location of the animal was detected by sensors and recorded automatically.

Light (10 W) was used as a conditional stimulus 5 s prior an unconditioned stimulus—a foot shock (0.4 mA, 5 s). The intertrial interval was 30 s. Training consisted of 5 days, each day a 50-trial avoidance sessions were performed. Each trial was classified as an avoidance response (crossing to the other side before the onset of the shock), an escape response (crossing to the other side after the onset of the shock), or a null response (remaining in the original compartment and receiving 5 s of shock)^89^. The results are presented as an average number of avoidance reactions per day per group.

#### Startle reflex, prepulse inhibition (PPI), habituation

Startle reflex and habituation served as a measure of sensorimotor gating, PPI was used to detect schizophrenia-related traits. The startle reflex, PPI and habituation were evaluated using an SR-Pilot device consisting of a plastic chamber with build-in piezoelectric sensors for movement detection and a controlled source of sound. The session consisted of 5 blocks of 64 trials in total as follows:

*Block 1, acclimation trials 1–6*: 5 min acclimation at background noise level (65 dB);

*Block 2, trials 1–6:* six pulse-alone trials (120 dB, 40 ms);

*Block 3, trials 7–32:* 26 pulse-alone trial (120 dB), prepulse (either 69, 73 or 81 dB) + pulse alone trial (120 dB), and NOSTIM trials in a pseudorandom order;

*Block 4, trials 33–58:* 26 pulse-alone trial (120 dB), prepulse (either 69, 73 or 81 dB) + pulse alone trial (120 dB), and NOSTIM trials in a pseudorandom order;

*Block 5, trials 1–6:* six pulse-alone trials (120 dB, 40 ms).

PP73 represented prepulse (73 dB, 20 ms) + pulse-alone trials (120 dB, 40 ms), PP81 represented prepulse (81 dB, 20 ms) + pulse-alone trials (120 dB, 40 ms), and NOSTIM trials represented record-only trials. The prepulses were presented 100 ms before the onset of pulse-alone stimuli. The inter-trial intervals lasted for about 15 s.

The average value for pulse alone in the blocks 3 and 4 were used to calculate Startle reflex. The difference between average values for pulse alone in blocks 2 and 5 were used to calculate Habituation. The difference between average values for pulse alone and prepulse + pulse in the blocks 3 and 4 were used to calculate PPI^90^:

$${\text{Startle}}\;{\text{ reflex}}\;\; \, = \;\;{\text{ average }}\;\left( {{\text{pulse}} - {\text{alone}}\;{\text{ in}}\;{\text{ blocks }}\;{\text{No}}\;{3 }\;{\text{and}}\;{ 4}} \right), \, \;{\text{expressed}}\;{\text{ in }}\;{\text{arbitrary }}\,{\text{units}};$$

$${\text{PPI}}\;\; = \;\;{1}00\; \times \;\left[ {{\text{pulse}} - {\text{alone}}\left( {{\text{block No3}},{4}} \right){-}\left( {{\text{prepulse }} + {\text{ pulse}}} \right)} \right]/{\text{pulse}} - {\text{alone}}\left( {{\text{blocks }}\;{\text{No}}\;{3}\;{\text{ and}}\;{ 4}} \right), \, \;{\text{expressed}}\;{\text{ in }}\;\% ;$$

$${\text{Habituation}}\; = \;{1}00 \times \, \left[ {{\text{pulse}} - {\text{alone}}\left( {{\text{block}}\;{\text{No}}\;{2}} \right) {-}{\text{ pulse}} - {\text{alone}}\left( {{\text{block }}\;{\text{No}}\;{5}} \right)} \right]/{\text{pulse}} - {\text{alone }}\left( {{\text{block}}\;{\text{No}}\;{2}} \right),\;{\text{expressed}}\;{\text{ in}}\; \, \% .$$

#### Social preference test

Social preference test demonstrates a test animal’s preference towards a live object rather than an unanimated one and can reflect autistic-like behavior. The social preference test was conducted according to the protocol described by Malkova with co-authors^91^ under red light in a rectangular test arena (60 cm × 40 cm) separated into three equal chambers (20 cm × 40 cm, left, central and right) with a 6 cm × 6 cm openings. An animal was placed in the central chamber and was allowed to move within the arena for 5 min for acclimation. An unfamiliar BALB/c male mouse was placed in one side chamber under a wire cup, whereas a white cotton ball of comparable size was placed under similar cup into the opposite chamber. Time spent in each chamber was analyzed automatically using Ethovision XT10 software. Social preference coefficient was calculated as (time spent in the “animal” chamber)/(time spent in the “cotton” chamber). Choosing a chamber to place a BALB/c mouse was random.

#### Odor preference test

Odor preference test allows evaluating animal's interest to odors without direct interaction with the odor source. BALB/c female and male bedding samples were placed in two separate tea infusers (Ikea, art. #469.568.00) and introduced into the test animal’s cage for 5 min. BALB/c animals were kept in their cages for a week before the test in order to soil the bedding. Characteristic nose and whisker movements were scored as “sniffing” and analyzed manually by two independent observers. The results are given as (time spent sniffing a specimen)/(total sniffing time) and expressed as percentages.

#### Resident-intruder tests

These tests were used to investigate social interest and social interactions in animals like aggression and courtship behavior. Test males were single-housed for at least 3 days prior to the test. Three month-old sexually experienced BALB/c animals were used as intruders. Either a female, a male or both intruders were placed in the test animal’s homecage for 15 min^92,93^, and social interactions were recorded by a video camera. Attacks and matings were counted manually during the test by two independent observes and averaged out. Duration and quantity of sniffing and licking was scored manually using video recordings by the two independent observes and averaged out. The female intruder test was performed at first followed by the male intruder test, and the two intruders test was performed last. The inter-test time was at least 2 days for each animal.

#### Metagenomic analysis

For the gut metagenomic analysis, intestinal contents (the content of small intestine, cecum, colon and a fecal sample) were collected in Inhibitex buffer, and total DNA was extracted using QIAamp DNA Stool Mini Kit according to manufacturer’s instructions (*n*(*C57BL/6*) = 4, *n*(*Muc2*^+*/*+^) = 4, *n*(*Muc2*^*−/−*^) = 4). Metagenomic analysis was performed by a commercially available service at Novogene (https://en.novogene.com) as follows. The *16S rRNA* V3-V4 region was used for barcoded amplification using Phusion High-Fidelity PCR Master Mix, and PCR-products were mixed in equal ratios. The mixture was purified with Qiagen Gel Extraction Kit. The libraries were generated with NEBNext UltraTM DNA Library Prep Kit for Illumina sequencing and analyzed by the Illumina platform. Data analyses were performed using Uparse software. Sequences with ≥ 97% similarity were assigned to the same operational taxonomic units (OTUs). Beta diversity analysis was used to measure differences between samples in terms of species complexity. Beta diversity of both: weighted and unweighted unifrac were calculated by QIIME software (Version 1.7.0). Principal component analysis (PCA) preceded cluster analysis to reduce the dimension of the original variables using the FactoMineR package and ggplot2 package in R software (Version 2.15.3). Differences in taxa abundance between groups were shown for the average top ten taxa in each group using normalized reads for each OTU. PCA analysis was performed using top 70 genera, identified in at least one of the test groups, top two PCs in terms of variance coverage were used for data plotting and identification of significant differences among the groups. The data is available at https://data.mendeley.com/datasets/y85tx8hzdh/draft?a=5fde3ad3-1078-4911-9523-92a361805999.

#### Metabolite extraction and nuclear magnetic resonance (NMR) spectroscopy

The metabolomic composition of the sample was analyzed at the Center of Collective Use «Mass spectrometric investigations» SB RAS (Novosibirsk, Russia) by high-resolution^1^H NMR spectroscopy. The extraction of metabolites from serum was performed by using a short sample preparation protocol earlier evaluated for quantitative NMR-based metabolomics^45,94^. Namely, 100 μl of ice-cold methanol and 100 μl of ice-cold chloroform were added to 100 μL of serum and vortexed for 30 s, kept on ice for 10 min, and incubated at − 20 °C for 30 min. The mixtures were centrifuged at 12,000 rpm and at 4 °C for 30 min to pellet proteins. The top hydrophilic fraction was collected to fresh vials and lyophilized using vacuum concentrator.

To obtain protein-free extract of metabolites from the mouse brain, we used the following sample preparation protocol earlier evaluated for quantitative NMR-based metabolomics^95^. Briefly, brains were collected in 1.5 ml Eppendorf tubes and frozen in liquid nitrogen. Frozen brain tissue was weighed and homogenized using a glass homogenizer in cold (− 20 °C) water/methanol/chloroform mixture in a ratio 1:2:2 (v/v; 1600 µl of solvent mixture per 150 mg of wet tissue), vortexed for 30 s, kept on ice for 10 min, and incubated at − 20 °C for 20 min. The mixtures were centrifuged at 12,000 rpm, 4 °C for 30 min to pellet proteins. The top hydrophilic fraction was collected to fresh vials and lyophilized using vacuum concentrator.

Dried extracts were re-dissolved in 600 µL of D2O containing 6 × 10^−6^ M DSS as an internal standard and 20 mM deuterated phosphate buffer to maintain pH 7.4. The^1^H NMR measurements were carried out with the use of the AVANCE III HD 700 MHz NMR spectrometer equipped with a 16.44 T Ascend cryomagnet. The proton NMR spectra for each sample were obtained with 64 accumulations. Temperature of the sample during the data acquisition was kept at 25 °C, the detection pulse was 90 degree. The repetition time between scans was 25 s to allow for the relaxation of all spins. Low power radiation at the water resonance frequency was applied prior to acquisition to presaturate the water signal. The pulse sequence zgpr was applied.

The collected NMR spectra were manually phased and baseline corrected. Signal processing and integration was performed using MestReNova V.12 software. We used DSS at a concentration of 6 µM as reference for chemical shift and determination of the metabolite concentration. The metabolite resonance assignments were made by comparison with data of Human Metabolome Database^96^ (http://www.hmdb.ca) and our own experience in the metabolomic profiling^97–100^, or by adding reference compounds whenever needed. The concentration of metabolites in the samples was calculated by the integration of the peak area of a metabolite respectively to DSS added to the sample. Amino acids, peptides, organic acids, alcohols, nucleotides, osmolytes, energy metabolism products were identified. The detailed description of metabolite quantification and validation are presented in our previous work^95^. The data is available at amdb.online/amdb/experiments/152/ (blood serum profiles), and amdb.online/amdb/experiments/154/ (brain profiles).

#### Real-time PCR

Fecal DNA was purified from fecal pellets using QIAamp DNA Stool Mini Kit according to the manufacturer’s recommendations. Bacterial abundance was measured by real-time PCR using BioMaster HS-qPCR SYBR Blue, 5 µL of fecal DNA and 300 nM specific primers (Supplementary Table 2). The data was normalized to *16SrRNA* as ∆Ct = 2^(Ct_*16SrRNA*_−Ct_bacterium of interest_) and shown as log_10_(∆Ct)^45^. For samples after antibiotic treatment, the data was normalized to *28SrRNA* as ∆Ct = 2^(Ct_*28SrRNA*_−Ct_bacterium of interest_) and shown as log_10_(∆Ct) or log_2_(∆Ct). For gene expression analyses, total RNA from colonic samples was purified using TRIzol reagent. One µg of RNA was used in reverse transcription reaction, cDNA was synthesized using M-MuLV reverse transcriptase according to the manufacturer’s recommendations. The reaction volume of 20 µL was then diluted up to 100 µL with deionized water and used for PCR. Real-time PCR reaction was prepared using a BioMaster HS-qPCR SYBR Blue, 5 µL of cDNA, and 250 nM specific primers. Gene expression was normalized to *Tubb5* mRNA as ∆Ct = 2^(Ct_*Tubb5* mRNA_ − Ct_gene of interest mRNA_).

#### Drug treatments

Vancomycin was diluted in drinking water at a concentration of 0.5 g/L. The animals of the experimental group (C57BL/6+VR) received vancomycin solution in drinking water for 2 weeks, rifampicin at a concentration of 0.075 g/L has been added in the last 8 days. During the testing period, animals continued to drink vancomycin + rifampicin solution. Control animals C57BL/6 received regular drinking water. Feces for *A. muciniphila* detection were collected directly on the mouse in three consecutive days starting the day of the first behavioral test.

Amoxicillin + clavulanic acid were diluted in drinking water at a concentration of 0.45 mg of amoxicillin and 0.15 mg of clavulanic acid per ml of water. *Muc2*^+*/*+^ (*Muc2*^+*/*+^ + AMC) animals received this solution for 2 weeks and during the testing period. Feces for *A. muciniphila* detection were collected directly on the mouse in three consecutive days starting the day of the first behavioral test. *Muc2*^*−/−*^ animals were also used for antibiotic treatment, but were withdrawn from this experiment due to excessive weight loss and high death rate.

Strychnine was administered intraperitoneally (i.p.) to *Muc2*^*−/−*^ animals (*Muc2*^*−/−*^ + strychnine) 15 min before the test^101^ at a dose of 0.75 mg/kg (the dose was selected empirically according to the published data^101,102^), in a volume of 10 ml/kg. Control animals were injected with the same volume of saline. The interval between the tests was 3 days.

The selective antagonist of the NMDA receptor glycine site L‐701,324 was dissolved in 99% saline, 1% DMSO (vehicle) and injected i.p. 45 min before the behavioral test in a dose of 10 mg/kg. Control mice received 1% DMSO in a volume of 10 mL/kg. The interval between the tests was 3 days.

#### Histological and clinical scores

Paraffin sections (4 µm) were stained with H&E (Hematoxylin and Eosin) stain. The sections were examined in a blinded manner. Hyperplasia was defined as the percentage of cells per crypt above the control. Erosion was defined as the area of epithelium where the crypt structure was lost and shown as the percentage per section. Hyperplasia scoring: 0, < 10%; 1, 10–50%; 2, 51–100%; 3, > 100%). PMN cell infiltration scores: 0, none; 1, mild; 2, moderate; 3, severe). Scores for erosion (percentage of area involved): 0, < 1%, 1, 1–15%, 2, 16–30%, 3, 31–45%, 4, 46–100%). The maximum score (Total score) that could result from this scoring was 10. For clinical scores, animal’s weight, stool consistency and fecal blood were evaluated manually. Weight loss scoring: 0, none; 1, 0–17%; 2, 18–35%; 3,> 35%. Stool consistency and fecal blood scoring: 0, normal droppings; 1, loose droppings; 2, diarrhea and 0, no blood; 1, visible blood in rectum; 2, visible blood on fur. The total score reflects the sum of all scores.

### Data analysis and statistics

#### Statistical analysis

The data are presented as mean ± stadard error of mean, bacterial PCR is shown as data points. The data were tested for normality using the Kolmogorov–Smirnov test. Normally distributed data were analyzed by Student’s t-test test for independent samples. Data involving multiple factors were analyzed using analysis of variance (ANOVA) followed by Fisher LSD test. Not normally distributed data were processed using Kruskal–Wallis test followed by Mann–Whitney *u*-test. Active avoidance was analyzed using Friedman test for repeated measures. Tail suspension results were analyzed using χ^2^ test. Principal component analysis (PCA) was performed in STATISTICA12 software, data significance in principal components (PC) was evaluated using ANOVA followed by Fisher LSD test (blood metabolomics) or using Kruskal–Wallis test followed by Mann–Whitney *u*-test (brain metabolomics and metagenomics). Correlation was analyzed by Pearson correlation analysis. The value of *p* < 0.05 was considered significant.

## Results

Behavioral phenotyping of *Muc2*^*−/−*^ male mice reveals high novelty-induced activity, reduced anxiety-related behavior, impaired startle reflex and abnormal social behavior.

First, we used behavioral phenotyping to investigate whether chronic inflammation in *Muc2* knockout model has a physiologically relevant effect on CNS. We evaluated behavioral pattern in the home cage environment using Phenomaster technology (Fig. 1A). Among parameters tested, *Muc2*^−/−^ mice showed a reduced motor activity and an increased water consumption rate as compared to C57BL/6. Most probably, increased drinking is a result of diarrhea in mutants. At the same time, locomotion was increased in a novel environment in *Muc2*^−/−^ males as revealed by the open field test (Fig. 1B). *Muc2*^−/−^ animals had increased horizontal activity at the level of a trend and significantly increased vertical activity (rearings). Moreover, *Muc2*^−/−^ mice spent significantly more time in the center of the arena indicating reduced anxiety. These results were supported by the light–dark test, where mutant animals showed an increased motor activity, more time and number of entries in the light compartment (Fig. 1C). Some reduction in anxiety was also noted in the elevated plus-maze where *Muc2*^−/−^ mice had an increased number of peeks down from the open arms (Fig. S1). However, there were no changes in the distance and time in open arms. In the marble burying test, *Muc2*^−/−^ animals buried significantly fewer marbles as compared to C57BL/6 indicative of the reduced repetitive behavior (Fig. 1D). Behavioral phenotyping revealed no depressive-like traits and a superior motor performance in *Muc2*^−/−^ mice (Fig. S1). We also found no impairment of cognitive functions, since there were no substantial differences in spontaneous alternation, long-term memory retention and active avoidance in mutants as compared to the control (Fig. S1). Next, we studied sensorimotor gating in *Muc2* model by measuring prepulse inhibition (PPI), startle reflex and habituation to the acoustic signal. We found no difference in PPI between C57BL/6 and *Muc2*^−/−^ animals, however, mutant mice had significantly weaker startle reflex and diminished habituation to the sound (Fig. 1E). Thus, colitis affected neuronal circuits responsible for sensorimotor gating in *Muc2*^−/−^ mice.

Social preference test revealed that *Muc2* mutation did not result in autistic phenotype, since mutant mice preferred an animal to an unanimated object in a social preference test (Fig. S1). However, in contrast to C57BL/6 animals, mutant males lack preference towards female intruders and tend to interact equally with a male and a female intruder in a two intruders paradigm (Fig. 1F). Control males strongly preferred to contact a female than a male, whereas mutant mice demonstrated no preferences towards either. Moreover, *Muc2*^−/−^ males interacted with a male intruder significantly more than did the control animals, revealing a substantial shift in social preference. These results were supported by the resident-intruder test with a male mouse, where *Muc2*^−/−^ males made more contacts to the intruder, with more animals exhibiting aggression or even mating to males (Fig. S2). At the same time, when a female mouse was used as an intruder, mutant males mated females at a lower rate than did the wild type controls (Fig. S2). Likewise, in the two intruders test, mutant males were more likely to mate male intruders and attack females, without any behavioral discrimination between male and female intruders (Fig. S2). In order to test whether *Muc2* mutation affects perception of socially-relevant olfactory signals we performed an odor preference test. By providing test animals with bedding from female and male cages we found that mutant males discriminated male and female odors at the same rate as their control counterparts (Fig. 1G).

Altogether, the main behavioral traits characteristic to *Muc2* colitis model are elevated novelty-induced locomotion and exploratory activity, reduced anxiety-related behaviors, weakened startle response and lack of behavioral discrimination between male and female animals. Importantly, these results are mainly relevant for male mice, and the effect of *Muc2* mutation on female behavior is yet to be investigated.

Behavioral traits of *Muc2*^−/−^ mice are transferrable to the wild-type animals upon littermate co-housing.

Our *Muc2* model behavioral data unexpectedly demonstrate enhancement of exploratory activity and reduction in anxiety, which is generally not common in chronic intestinal inflammation. Thus, we suggested that the observed phenotype is attributed to the colitis-associated microbiota rather than inflammation per se. As mucosal environment strongly affects intestinal flora, we performed littermate co-housing of *Muc2*^−/−^ and C57BL/6 animals in order to facilitate microbiota exchange and test its involvement in the behavioral phenotype. C57BL/6 were crossed to *Muc2*^−/−-^ animals to generate heterozygous mutants, which were mated to obtain *Muc2*^+/+^ (wild-type littermates) test group (Fig. 2A). These animals had no features of intestinal inflammation or any clinical signs of colitis as evaluated by histological examination and inflammatory cytokine profiling (Fig. S3).

Interestingly, *Muc2*^+*/*+^ mice reconstituted many behavioral features of *Muc2*^−/−^ animals in the open field, light–dark, and marble burying tests (Fig. 2). In the open field test, *Muc2*^+*/*+^ mice showed significantly greater activity in comparison with C57BL/6, and reduced anxiety (Fig. 2B). In the light–dark test, *Muc2*^+*/*+^ animals were more active in the light compartment as compared to C57BL/6 (Fig. 2C). Elevated plus-maze revealed no differences between the test groups (Fig. S3). Startle reflex and habituation were on average reduced in *Muc2*^+*/*+^ animals, but these differences were not statistically significant (Fig. S3). At the same time, in the marble burying test, *Muc2*^+*/*+^ animals buried fewer marbles consistent with the decreased repetitive behavior and reduced anxiety in mutants (Fig. 2D). Social behavior in *Muc2*^+*/*+^ animals was also similar to that of the mutants: *Muc2*^+*/*+^ males made no preferences towards females in terms of social contacts, interacted with a male intruder more than C57BL/6 males, attacked females and mated male intruders (Figs. 2E, S3).

We then proposed that if the behavioral pattern in *Muc2*^+*/*+^ animals originates from the *Muc2*^*-/-*^ microbiota transfer, then the direct co-housing with the control C57BL/6 mice should minimize phenotypic differences between these groups. Thus, we co-housed genetically identical *Muc2*^+*/*+^ littermate progeny and C57BL/6 animals to equilibrate their microbiota and found no significant differences in the same panel of behavioral tests (Fig. S4). These data demonstrate that the behavioral traits in *Muc2*^−/−^ animals, at least in part, are attributed to the microbiota composition rather than intestinal inflammation itself. Probably, microbiota improves the deteriorating effect of colitis on CNS physiology and masks potential behavioral deficits in mutant mice.

Manipulation of intestinal microbiota affects behavioral traits associated with *Muc2* mutation.

**Figure 3:**
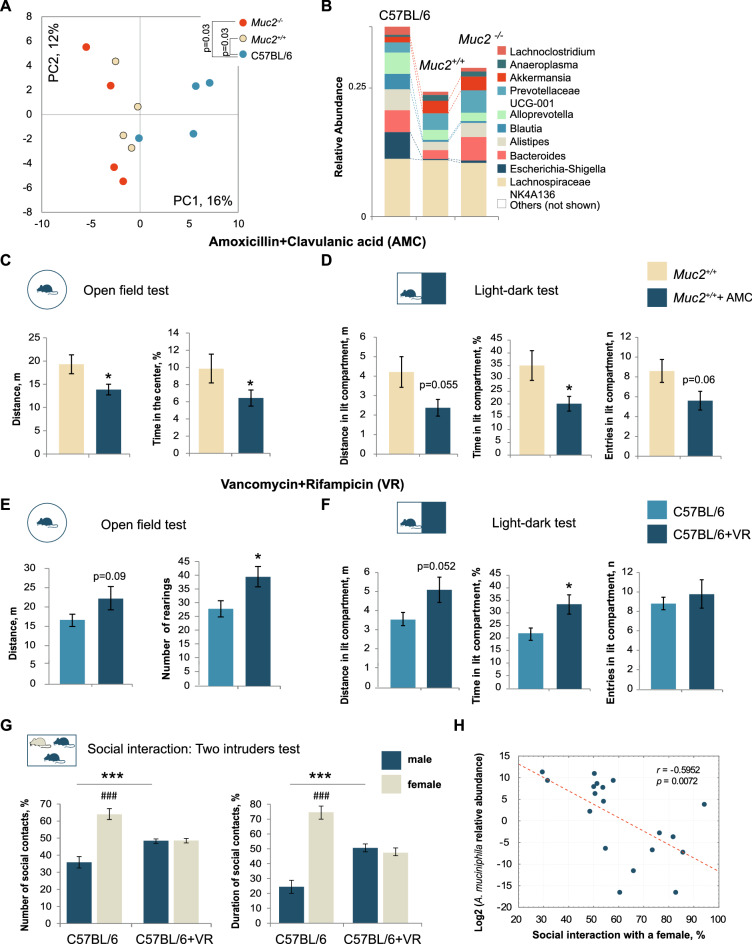
Intestinal microbiota defines behavioral phenotype in the *Muc2* mouse model of colitis. (**A**) PCA of metagenomic data based on top 70 abundant genera (*n* = 4). There was a significant effect of the group in PC1 (*p* = 0.044, Kruskal–Wallis test). There was a significant difference between C57BL/6 and *Muc2*^*−/−*^ and C57BL/6 and *Muc2*^+*/*+^ groups (*Muc2*^*−/−*^: Z = 2.16*, p* = 0.03; *Muc2*^+*/*+^: Z = 2.16, *p* = 0.03, Mann–Whitney *u*-test). (**B**) Qualitative changes in average relative abundance of top 10 abundant genera. (**C**) Open field test after AMC treatment (*n* = 10/group). Distance: *t* = 2.34, *p* = 0.03; rearings: *t* = − 1.82, *p* = 0.086; time in the center: Z = − 1.97, *p* = 0.049; Mann–Whitney *u* test. (**D**) Light–dark test after AMC treatment (*n* = 10/group). In the lit compartment: time: *t* = 2.30, *p* = 0.034; distance: *t* = 2.05, *p* = 0.055; entries: *t* = 2.008, *p* = 0.059, Student's *t*-test. (**E**) Open field test after VR treatment (*n* = 9/group). Distance: *t* = − 1.79, *p* = 0.09; rearings: *t* = *− *2.47, *p* = 0.025; time in the center: Z = − 1.97, *p* = 0.049; Mann–Whitney *u* test. (**F**) Light–dark test after VR treatment (*n* = 9/group). In the lit compartment: time: *t* = − 2.553, *p* = 0.022; distance: *t* = − 2.09, *p* = 0.052; entries: *t* = − 0.643, *p* = 0.529, Student's *t*-test. (**G**) Two intruders test after VR treatment (*n* = 9–10). Two-way ANOVA revealed a statistically significant interaction between the treatment and the intruder gender (number of contacts: *F*(1, 34) = 28.181, *p* < 0.001; duration: F(1, 34) = 56.854, *p* < 0.001. Number of contacts (C57BL/6, male *vs.* female): *p* < 0.001, Fisher’s LSD test; duration (C57BL/6, male *vs.* female): *p* < 0.001, Fisher’s LSD test; number of contacts with a male/female intruder *vs*. treatment: *p* < 0.001, Fisher’s LSD test; duration of contact with a male/female intruder *vs*. treatment: *p* = 0.001, Fisher’s LSD test. (**H**) Correlation analysis between social interaction with a female and relative abundance of *A. muciniphila* in the intestinal contents in the control and VR-treated animals: *r* = − 0.595, *p* = 0.007, Pearson’s correlation analysis. * = *p* < 0.05, ** = *p* < 0.01, *** = *p* < 0.001, *vs*. treatment. ### = *p* < 0.001, male *vs*. female.

We further characterized the intestinal bacterial community in mutant mice along with their wild-type littermates using new generation sequencing of the V3-V4 hypervariable region of *16S rRNA* gene. PCA analysis revealed that mutant animals had substantial differences in the intestinal bacterial community, which were passed via littermate microbiota transfer to the *Muc2*^+*/*+^ mice. Principal component analysis (PCA) revealed significant differences between C57BL/6 and *Muc2*^−/−^ and C57BL/6 and *Muc2*^+*/*+^ groups (Fig. 3A). Relative abundance of top 10 bacterial genera revealed a decrease in the proportion of *Blautia* and *Escherichia-Schigella*, and an increase in *Akkermansia* upon transfer of *Muc2*-associated microbiota (Fig. 3B). Real-time PCR using species-specific primers (Fig. S5) confirmed a significant overproliferation of *Akkermansia muciniphila*. To further test the interrelations between behavioral features and microbiota in *Muc2*^+*/*+^ mice, we treated these animals with a broad-spectrum antibiotic amoxicillin combined with a clavulanic acid (AMC) followed by the open field test and the light–dark test. A significant decrease in *A. muciniphila* in the intestine of *Muc2*^+*/*+^ animals was confirmed by real-time PCR (Fig. S5). Open field test showed that AMC treatment decreased motor activity and increased anxiety-related behavior (Fig. 3C). Light–dark test further confirmed that inhibition of microbiota in *Muc2*^+*/*+^ mice reduced exploratory activity (Fig. 3D). At the same time, clinical score in *Muc2*^+*/*+^ animals upon AMC treatment was 0 (Fig. S3), and no sickness behavior was observed. These results suggested that inhibition of the intestinal bacteria lead to the normalization of some behavioral traits associated with *Muc2* colitis model.

Next, we reasoned that the behavioral phenotype might be attributed to the abundance of *Akkermansia* as it was the only genus upregulated in *Muc2*^−/−^ and *Muc2*^+*/*+^ mice, and *A. muciniphil*a was significantly increased in *Muc2*^*-/-*^ as compared to C57BL/6 animals (Figs. 3B, S5). Thus, we aimed to mimic *A. muciniphila* overproliferation in C57BL/6 mice using vancomycin, as it was previously shown to elevate *Akkermansia*^41^. At the same time, this antibiotic is known for occasional strong outbreaks of *Escherichia coli* proliferation, which might also affect behavior^42–44^. We have shown previously that *E. coli* found in our in-house colony of C57BL/6 is particularly sensitive to rifampicin^45^, so we treated C57BL/6 mice with a combination of vancomycin and rifampicin (VR). PCR analysis revealed that this scheme of antibiotic treatment effectively upregulated *A. muciniphila* in the intestinal contents of C57BL/6 animals and reduced *E.coli* outbreaks (Fig. S5). Finally, we performed the open field, light–dark and two intruders tests to analyze the effect of *Akkermansia* expansion on exploratory activity, anxiety-related behavior, and social interactions. In the open field test, RV-treated animals tended to be more active (Fig. 3E). The mutant behavioral phenotype was also partially reconstituted in the light–dark test as VR treatment induced activity in the light compartment (Fig. 3F). Even more pronounced was the effect of VR on social behavior: VR-treated males lost preference towards females in the two intruders test, and they interacted with a male intruder more than the control males (Fig. 3G). Moreover, the time of interaction with a female negatively correlated with the abundance of *A. muciniphila* in the intestinal contents of the test animals (Fig. 3H). Altogether, these results suggest that *A. muciniphila* is a potential regulator of CNS physiology in *Muc2-*associated colitis.

*Muc2*-associated microbiota affects blood metabolomic profile and brain glycine level.

**Figure 4:**
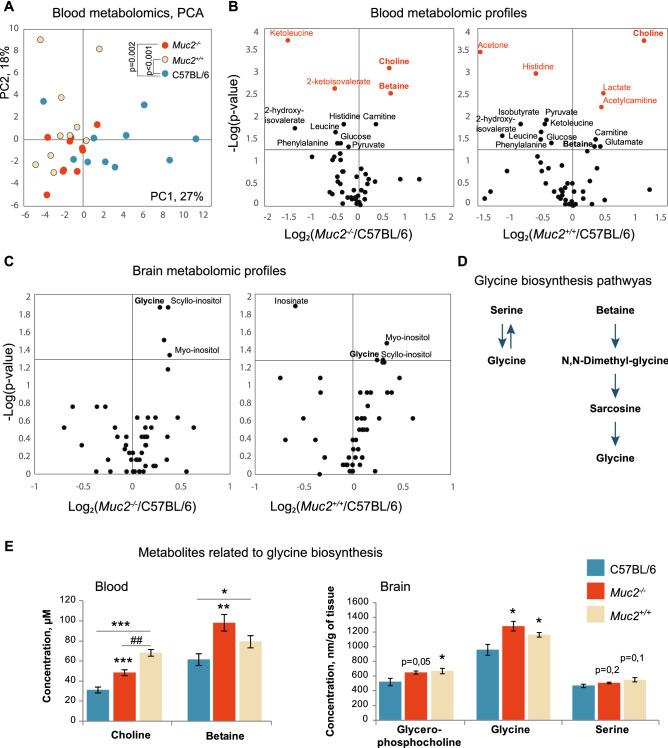
Metabolomic profiling of blood and brain of *Muc2*^*−/−*^ and *Muc2*^+*/*+^ mice. (**A**) PCA analysis of blood NMR metabolic profiles (*n* = 10/group). There was a significant effect of a group on PC1, which accounted for about 26.6% of variance (*F* (2, 27) = 8.84, *p* < 0.001, one-way ANOVA). Metabolomic profile of the C57BL/6 was significantly different from the two other groups (*Muc2*^*−/−*^: *p* = 0.002; *Muc2*^+*/*+^: *p* < 0.001, Fisher’s LSD test). (**B**) Volcano plots of blood metabolites as revealed by NMR. Horizontal line depicts a cut-off at *p* = 0.05. Vertical line depicts the ratio of 1. Metabolites with differences at *p* < 0.01 are shown in red. (**C**) Volcano plots of brain metabolites as revealed by NMR (*n* = 5–6). (**D**) The schemes of glycine biosynthesis from serine and betaine. (**E**) The levels of metabolites related to glycine biosynthesis. The data of the Kruskal–Wallis test followed by Mann–Whitney *u*-test are presented in Supplementary tables 3 and 4. * = *p* < 0.05, ** = *p* < 0.01 *vs*. C57BL/6. ## = *p* < 0.01 *vs*. *Muc2*^+*/*+^*.*

As microbiota transfer partially reconstitutes behavioral traits in the wild-type littermates in the absence of inflammation, we proposed that metabolomic changes induced by intestinal microbes might mediate the gut-brain interaction in the *Muc2* model of colitis. To test this hypothesis, we performed blood metabolic profiling in *Muc2*^−/−^, *Muc2*^+*/*+^ and C57BL/6 animals using NMR spectroscopy. PCA analysis revealed that *Muc2*^−/−^ littermate co-housing strongly affects host metabolism, and metabolomic profile of the C57BL/6 was significantly different from the two other groups (Fig. 4A). Metabolites with similar changes in *Muc2*^−/−^ and *Muc2*^+*/*+^ mice are more likely to be involved in regulation of *Muc2*-associated behavioral pattern. Among those were choline, betaine, ketoleucine, 2-hydroxy-isovaleriate, leucine, phenylalanine, glucose, and carnitine (Fig. 4B, Table S2).

To further investigate the effect of co-housing on brain metabolism, we performed NMR-based metabolomic profiling of brain tissue from *Muc2*^−/−^, *Muc2*^+*/*+^ and C57BL/6 animals. Based on 50 detected metabolites, PCA did not reveal any overall differences between the groups. However, there were significant changes in some metabolites in *Muc2*^−/−^ and *Muc2*^+*/*+^ groups in comparison to C57BL/6 (Fig. 4C, Table S3). Inositol (myo- and scyllo- isomers) and glycine were elevated in both test groups (Fig. 4C). Glycine was the most interesting among these as it is a neurotransmitter in the CNS and is involved in regulation of anxiety and sensorimotor gating. The major route of glycine biosynthesis is from serine via a reversible reaction catalyzed by Serine hydroxymethyltransferase. Alternatively, glycine can be synthesized from choline via betaine as an intermediate (Fig. 4D). Serine was slightly increased in the brain of *Muc2*^+*/*+^ animals, but no significant difference was found for this metabolite (Fig. 4E), whereas blood NMR profiles did not reveal a reliable serine signal. At the same time, both blood choline and betaine levels were higher in *Muc2*^−/−^ and *Muc2*^+*/*+^ animals (Fig. 4E). Brain NMR showed no differences in choline, whereas betaine was not detected (Table S3). However, there was an elevation of glycerophosphocholine in the brain (Fig. 4E). Thus, a potential cause of glycine upregulation in the brain might be blood betaine and choline, that are linked to bacterial metabolism^4^. Given the results of blood and brain NMR, we suggested that glycine might be a potential central mediator of the behavioral phenotype observed in *Muc2* colitis model.

Pharmacological inhibition of glycine neurotransmission normalizes behavioral changes in *Muc2*^−/−^ mice.

**Figure 5:**
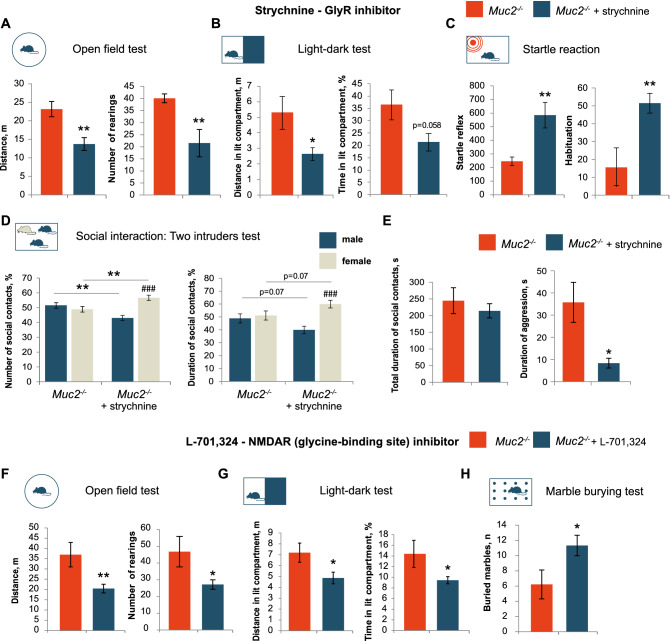
Glycine neurotransmission mediates behavioral abnormalities in *Muc2* model of colitis. (**A**) Open field test after strychnine treatment (*n* = 10–13). Distance: *t* = 3.53, *p* = 0.001; rearings: *t* = 2.897, *p* = 0.009, Student's *t* -test. (**B**) Light–dark test, (*n* = 7–8). Distance in light compartment: *t* = 2.21, *p* = 0.045; time in light compartment: *t* = 2.08, *p* = 0.058, Student's *t*-test. (**C**) Startle reflex and habituation (*n* = 10–12; startle: *t* = − 3.16, *p* = 0.005; habituation: *t* = − 3.09, *p* = 0.006, Student's *t*-test). (**D**) Two intruders test (*n* = 10/group). Two-way ANOVA revealed a statistically significant interaction between the treatment and the intruder gender (number of contacts: *F*(1, 46) = 21.39, *p* < 0.001; duration: *F*(1, 46) = 6.95, *p* = 0.011. Number of contacts (*Muc2*^*−/−*^ + strychnine, male *vs.* female, *p* < 0.001, Fisher’s LSD test; duration (*Muc2*^*−/−*^ + strychnine, male *vs.* female): *p* < 0.001, Fisher’s LSD test. Number of contacts with a male/female intruder *vs.* treatment, *p* = 0.003, Fisher’s LSD test; duration of contact with a male/female intruder *vs*. treatment, *p* = 0.07, Fisher’s LSD test. (**E**) Two intruders test (*n* = 10/group). Total duration of social contacts (not significant), duration of aggression: *t* = 0.75, *p* = 0.022, Student's *t*-test. (**F**) Open field test after L-701,324 treatment (*n* = 5–11). Distance: *t* = 3.303, *p* = 0.005; rearings: *t* = 2.71, *p* = 0.017, Student's *t* -test. (**G**) Light–dark test, (*n* = 5–11). Distance in light compartment: *t* = 2.36, *p* = 0.033; time in light compartment: *t* = − 2.56, *p* = 0.023, Student's *t*-test. (**H**) Marble burying test (n = 9/per group, Z = − 1.99, *p* = 0.047; Mann–Whitney *u* test). * = *p* < 0.05, *** = *p* < 0.001 *vs*. C57BL/6. ### = *p* < 0.001, male *vs*. female.

To understand the role of glycine-dependent neurotransmission in regulation of the *Muc2* mouse behavior, we aimed to partially compensate its up-regulation by inhibiting strychnine-sensitive glycine receptors (GlyR) and glycine binding sites in *N*-methyl-d-aspartate receptors (NMDAR)—the well-known glycine targets. Glycine acts as an inhibitory neurotransmitter via GlyRs, and as a glutamate co-activator at NMDARs. First, we inhibited glycine binding sites at GlyRs via i.p. injection of their inhibitor strychnine to *Muc2*^−/−^ male mice. In the open field test, strychnine significantly reduced motor activity (Fig. 5A). Likewise, in the light–dark test, there was a decrease in anxiety (Fig. 5B). Moreover, strychnine normalized the startle reflex and habituation in mutant males (Fig. 5C). Most notably, GlyRs inhibition in *Muc2*^−/−^ mice restored social behavior in the two intruders test and reduced overall aggression with no significant effect on the total duration of social contacts (Fig. 5D, E). At the same time, strychnine did not affect marble burying behavior (Fig. S5).

To inhibit glycine neurotransmission at NMDA receptors, we used a specific inhibitor of their glycine-binding sites, L-701,324. In the open field test, L-701,324 caused a decrease in activity (Fig. 5F). This result was supported by the light–dark test as L-701,324-treated animals spent less time investigating the light compartment (Fig. 5G). Interestingly, L-701,324 also normalized marble burying: *Muc2*^−/−^ animals buried more marbles upon administration of L-701,324 (Fig. 5H). However, L-701,324 did not improve social behavior and startle reflex (Fig. S5), which indicates the selective role of both receptors in the complex behavioral phenotype observed in *Muc2* knockout mice. Taken together, both inhibitors ameliorated all behavioral effects related to novelty-induced hyperactivity, anxiety, sensorimotor gating, and social behavior. Therefore, glycine appears to be the major microbiota gut-brain axis regulator in the mucus deficiency model of colitis.

## Discussion

*Muc2* knockout mouse model recapitulates key features of chronic intestinal inflammation and demonstrates high clinical and histological score of colitis^46^. At the same time, our behavioral phenotyping revealed no signs of anxiety- or depression-related behavior, as was found previously in other IBD models and human studies^25,27,28,47^. This finding suggests that microbiota defines the mutant behavioral profile. Indeed, microbiota transfer via littermate co-housing and combination of antibiotic treatments supported this hypothesis.

Identification of bacterial species associated with mutant behavioral traits revealed an increase in *A. muciniphila*. Interestingly, *A. muciniphila*, the major *Akkermansia* species in the intestine*,* is a mucin-degrading species and is usually associated with healthy gut and positive effects on the host physiology^48,49^. Its up-regulation in *Muc2* knockout seems discrepant given the nature of this mutation. Probably, the decrease in other commensal bacterial species like *Escherichia* and *Blautia* provide a niche to *Akkermansia* in the gut. At the same time, a number of membrane-bound mucins are present throughout the GI tract^50^. It is possible that the lack of Muc2 increases access to other mucins, which might be a preferred mucus source to *Akkermansia*. Consistent with our findings, published data indicate that probiotic treatment with *A. muciniphila* reduces depression, anxiety-related and repetitive behavior and ameliorates motor degeneration, learning and memory deficits in various mouse models^51–53^. At the same time, *Akkermansia* has not been shown to regulate male-male or male–female behaviors, like aggression and courtship. Here we show that intestinal microbiome strongly affects these behaviors, with *A. muciniphila* emerging as a key player in male-male and male–female social interactions.

Metabolic regulation represents one of the major possible communication routs in the microbiota gut-brain axis. Our NMR-based metabolomics shows that blood metabolic profiles shifts along with *Muc2*-associated microbiome transfer via littermate co-housing, supporting a significant impact of microbiota on host metabolism noticed earlier^54,55^. Brain metabolomic profiles, even though did not show such a drastic association with microbiota, revealed glycine as a potential neurotransmitter responsible for the observed behavior. In our model, inhibition of the glycine binding sites at Gly and NMDA receptors normalized the behavioral profile of *Muc2* knockout animals (Fig. 5). Glycine was previously shown to mitigate anxiety-related behaviors, ameliorate obsessive–compulsive disorder, and control sensorimotor gating^56,57^. Most probably, anxiolytic effect of glycine partially masks the effect of inflammation on behavior underlying the lack of depression- and anxiety-like phenotypes in *Muc2* chronic inflammation model. A notable addition to the already known behavioral repertoire of glycine is the involvement of GlyRs in male-male and male–female interactions.

An important question remains on the mechanisms of the central glycine up-regulation and its relation to *A. muciniphila*. Blood metabolomic profiling revealed elevation of choline and betaine in both *Muc2*^−/−^ and *Muc2*^+*/*+^ mice. Choline and betaine are precursors of glycine biosynthesis in the glycine-betaine pathway^58,59^. It is possible that elevated levels of these metabolites define high brain glycine. Choline was shown to readily cross the blood–brain barrier^60^ and reduce anxiety-related and repetitive behavior in a mouse model of autism^61^. Similar effects were shown for the choline downstream metabolic product—betaine^62–64^. These effects resemble the phenotype observed in *Muc2* model, and therefore elevated choline and betaine might mediate the gut-brain crosstalk. There are a number of potent ways for choline to affect CNS function, including up-regulation of the brain acetylcholine^65,66^. We have not investigated this neurotransmitter and its effects here as elevated pre-synaptic choline was shown to impair spontaneous alternations in Y-maze and reduce time in open arms in elevated plus-maze^67^, which is not consistent with our behavioral phenotyping. Moreover, we did not observe choline up-regulation in the brain indicating its rapid peripheral metabolism, which is consistent with the increased blood betaine.

In turn, free choline either comes from the diet or is released by phospholipase D from phosphatidylcholine, known as a main choline depot^68,69^. A possible mechanism might involve a shift in the intestinal phospholipid metabolism induced by *Akkermansia* via Toll-like receptor 2^70^. In support of this hypothesis, *Muc2* knockout mice were shown to have deregulated energy pathways and activated lipid metabolism^71,72^. So far, it is unclear whether metabolic features of mucin-deficient animals shape their microbial community so that microbiota further promote these changes in a positive feedback manner, or the lack Muc2 predisposes to the microbial changes first. It would be important to investigate this line of relations further, as it might give a possible target in microbiota-induced metabolism to combat neurological consequences of colitis.

## Supplementary Information

Supplementary Information.

## Data Availability

All data reported in this paper will be shared by the lead contact (kozhevnikovaen@physiol.ru) upon request.
