# Supplementary material for: Colitis-associated intestinal microbiota regulates brain glycine and host behavior in mice
Source: Sci Rep. 2022 Sep 29;12:16345. doi: 10.1038/s41598-022-19219-z (PMC9522854; doi:10.1038/s41598-022-19219-z)
Supplement: Supplementary file 1 — Supplementary Information. [file 41598_2022_19219_MOESM1_ESM.pdf]

Supplementary figure 1

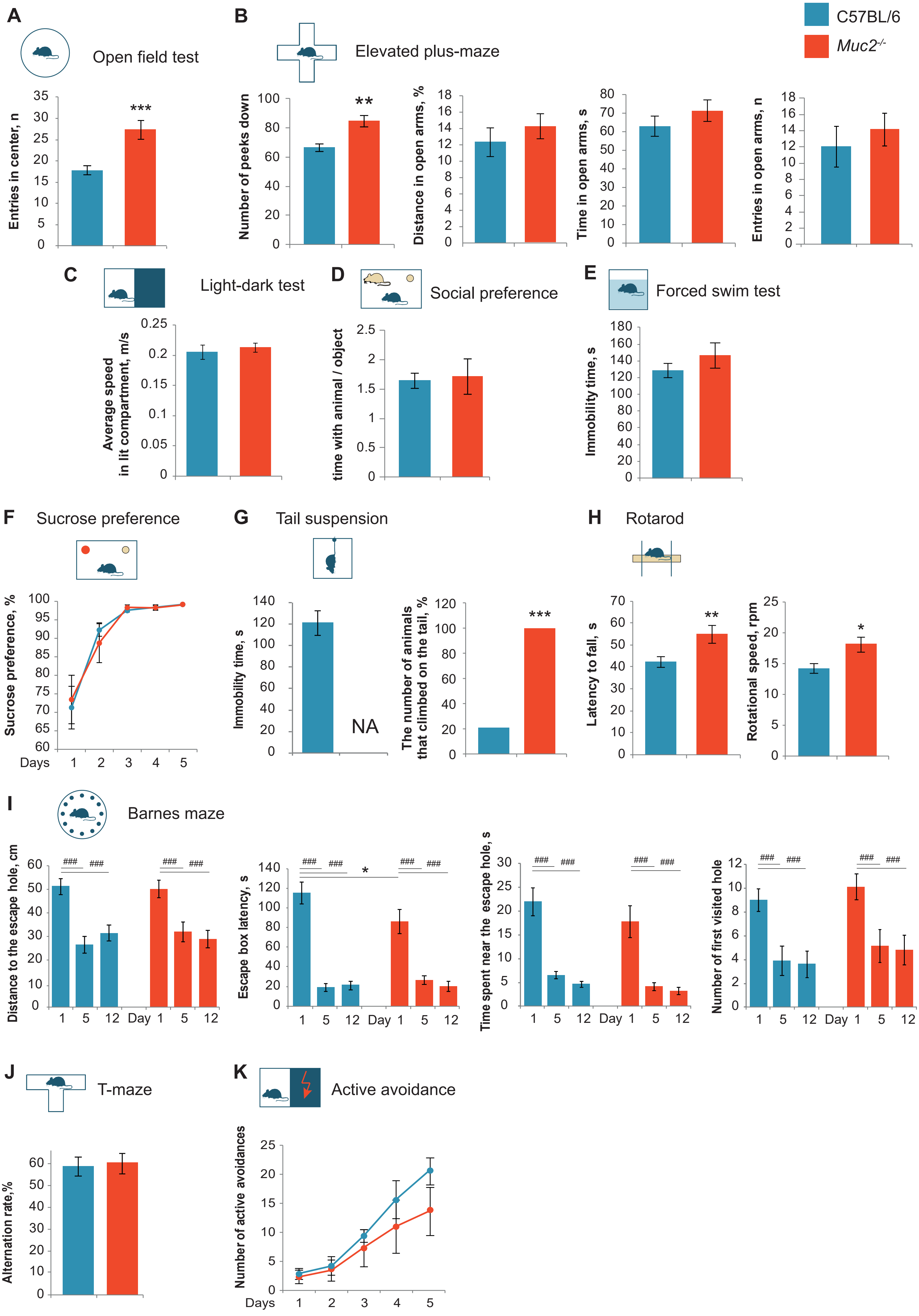

Supplementary figure 2

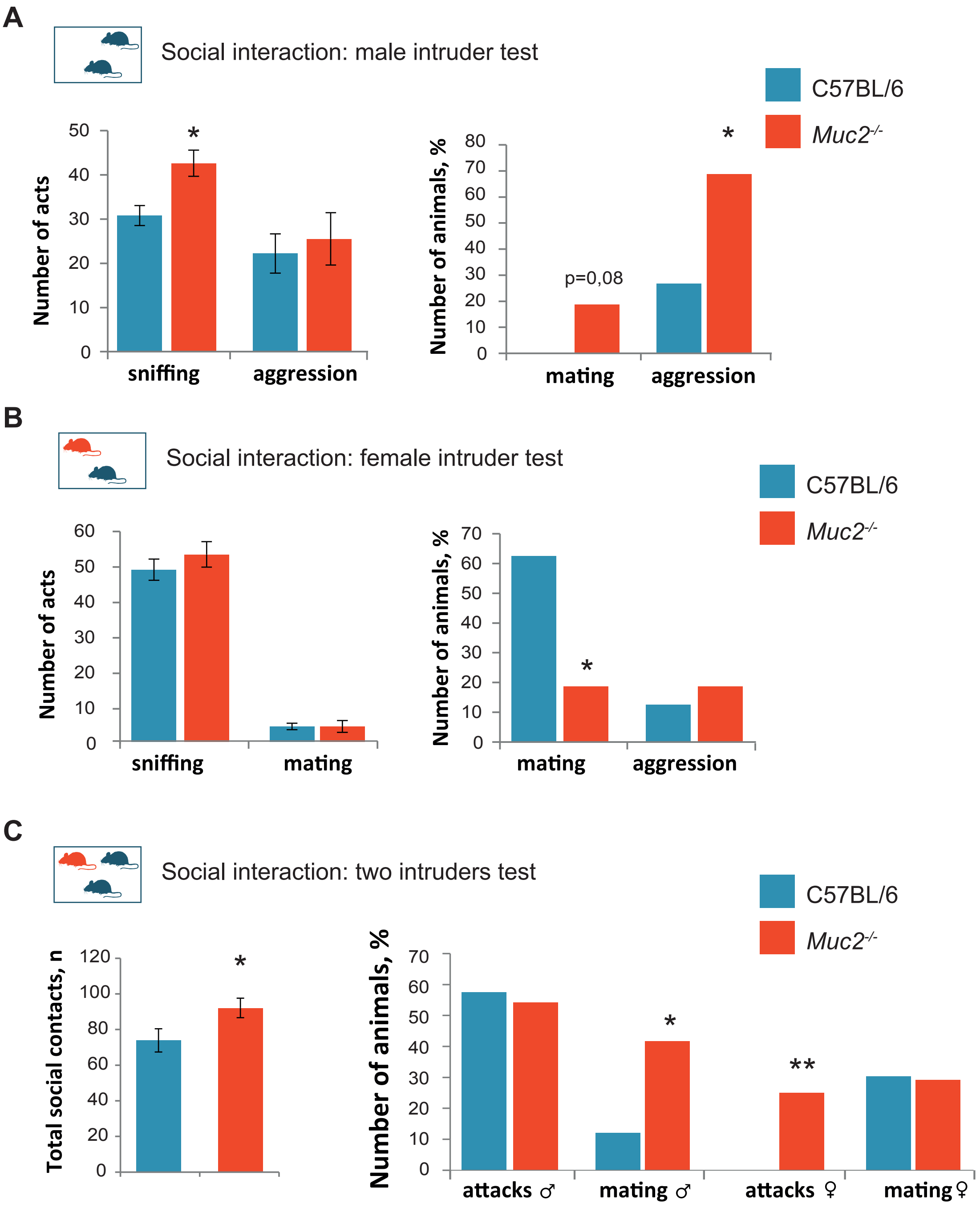

Supplementary figure 3

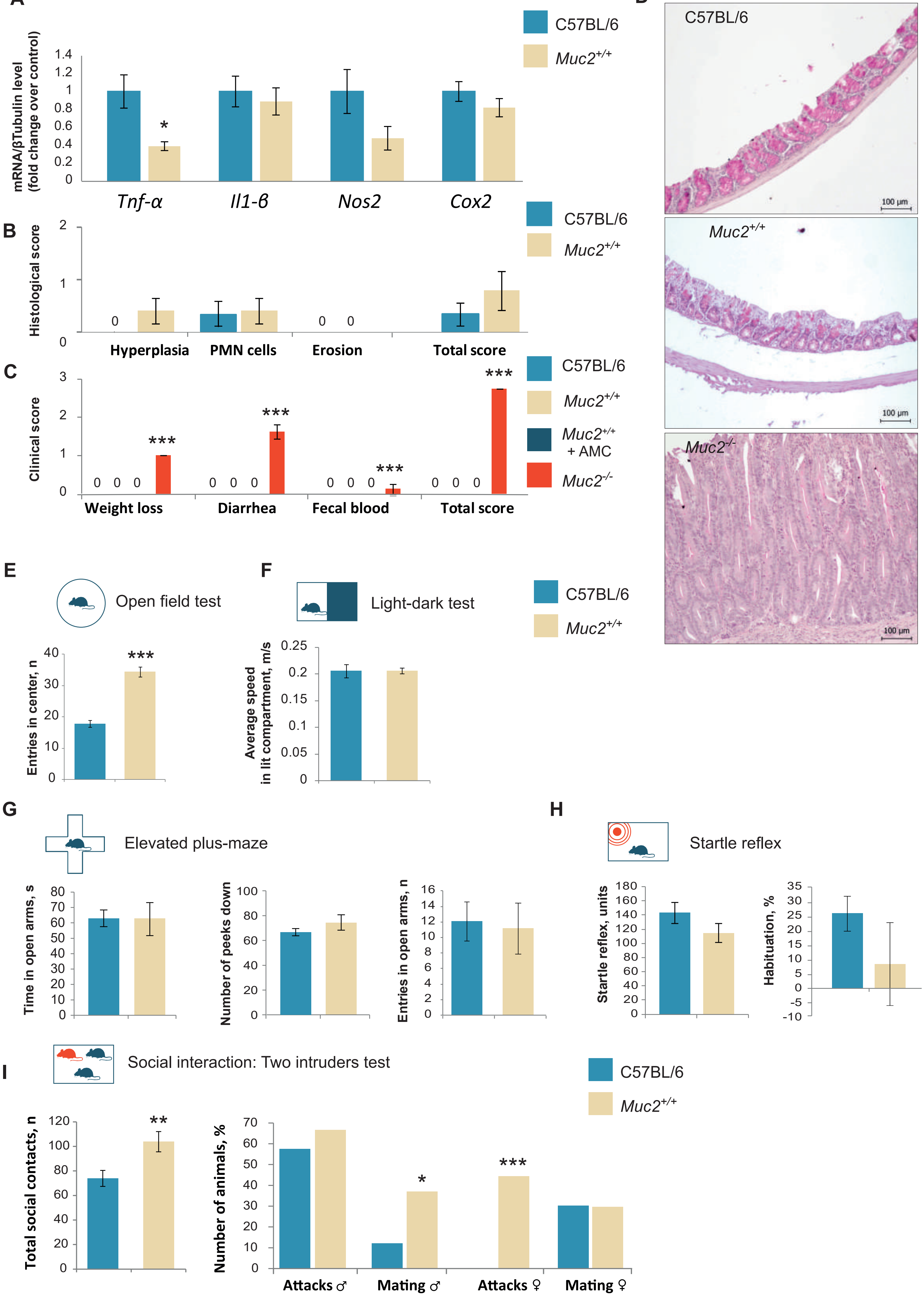

Supplementary figure 4

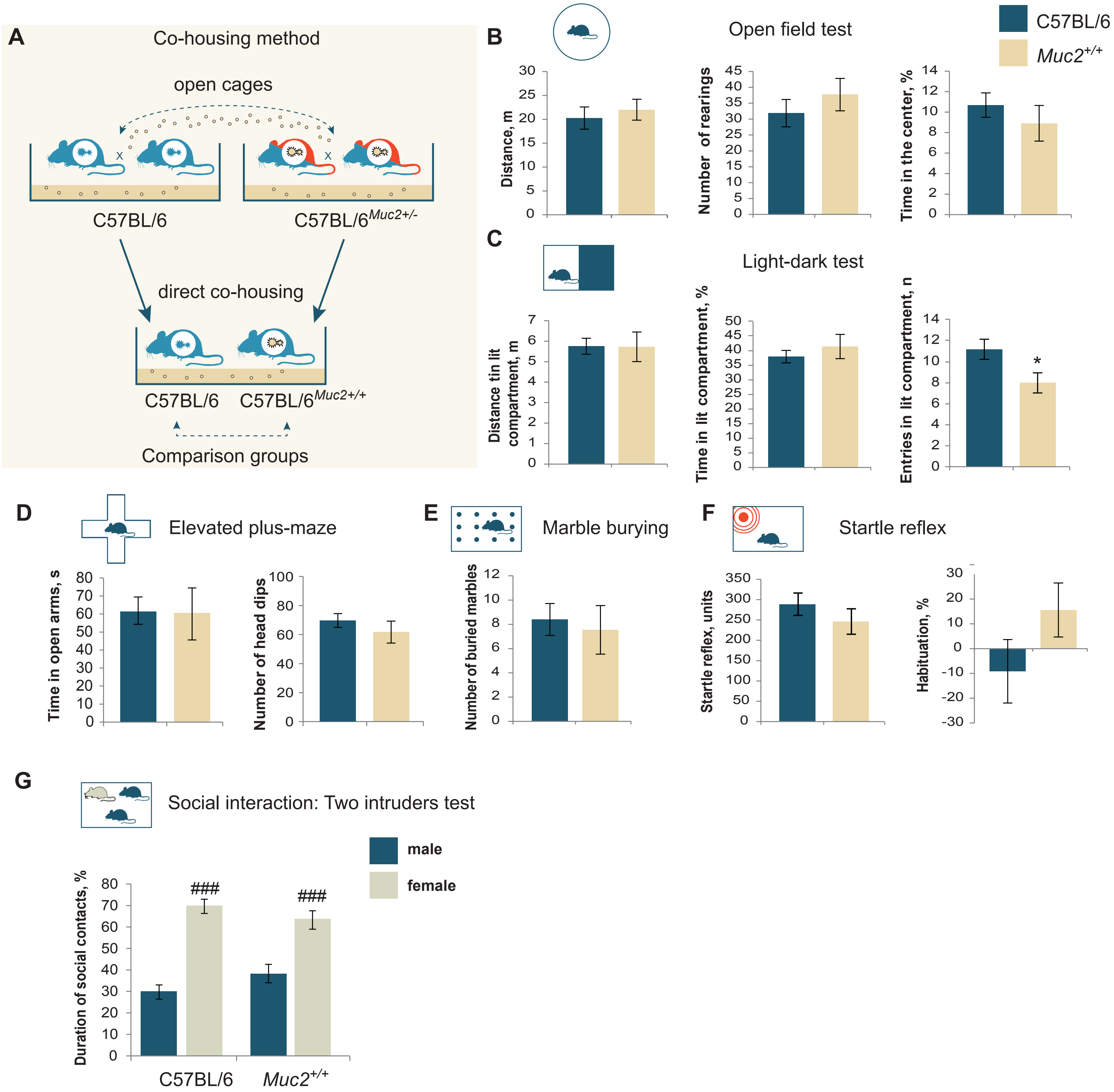

Supplementary figure 5

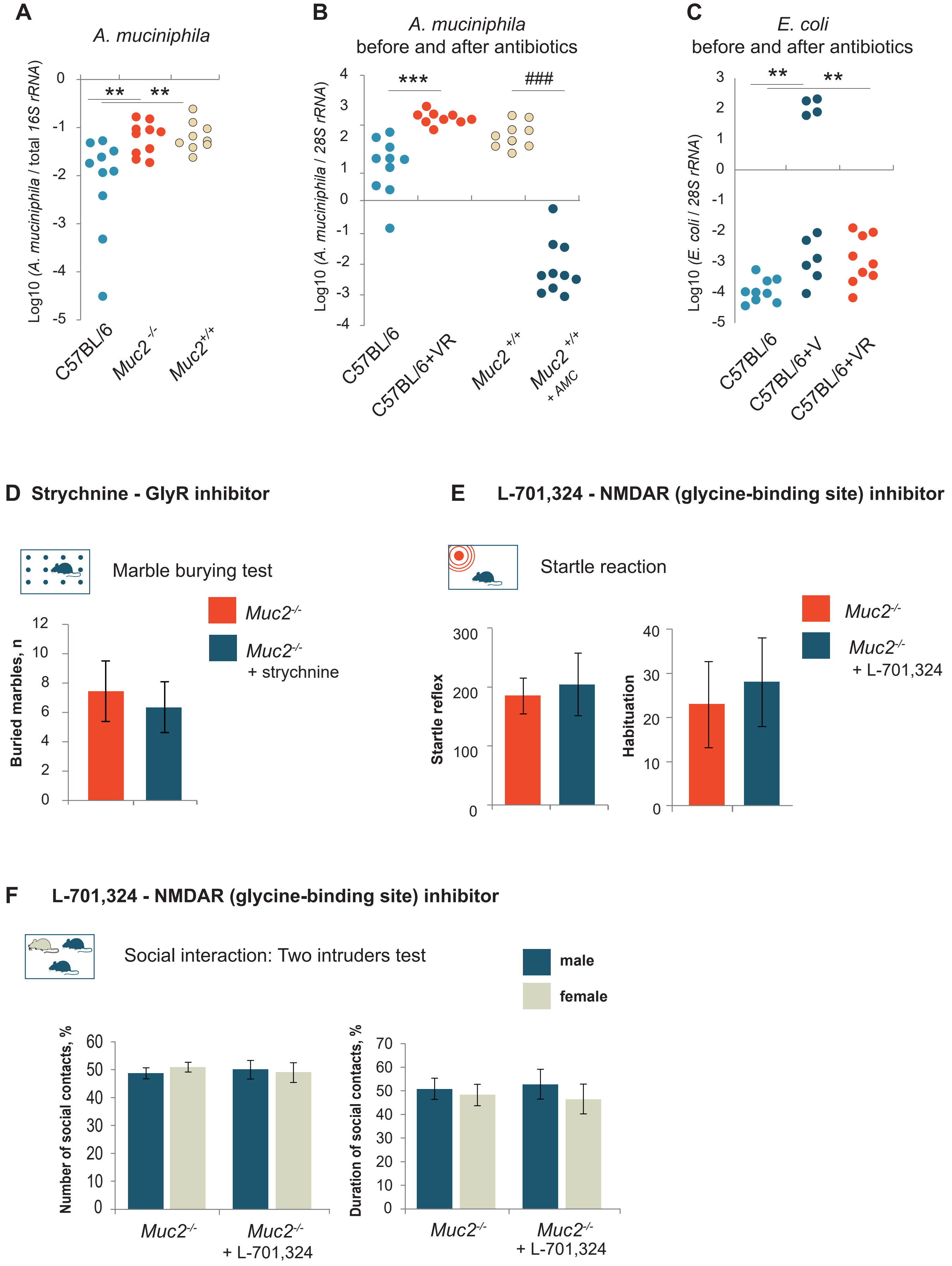

Supplementary Table 1 (Table S1)		
**The timeline of behavioral experiments with *Muc2* knockout mice and control C57BL/6 mice.**		
Open field test, Light-dark test, Elevated plus-maze, Marble burying test, Odor preference test, Rotarod test, Barnes maze, Social preference, Two intruders test, Male intruder test, Female intruder test, Habituation and startle reflex were tested in two trials using different groups of animals. The results of the trials were combined.		
**Group**	**Day**	**Test**
1	1	Open field test
	5	Light-dark test
	9	Elevated plus-maze
	13	Marble burying test
	17	Odor preference test
	21	Rotarod test
	25	Barnes maze. Habituation
	26	Barnes maze. Training
	27	Barnes maze. Training
	28	Barnes maze. Training
	29	Barnes maze. Training
	30	Barnes maze. Testing
	36	Barnes maze. Long-term memory retention
	40	Tail suspension
	44	Active avoidance
	45	Active avoidance
	46	Active avoidance
	47	Active avoidance
	48	Active avoidance
**Group**	**Day**	**Test**
2	1	Open field test
	5	Light-dark test
	9	Elevated plus-maze
	13	Marble burying test
	17	Odor preference test
	21	Rotarod test
	25	Barnes maze. Habituation
	26	Barnes maze. Training
	27	Barnes maze. Training
	28	Barnes maze. Training
	29	Barnes maze. Training
	30	Barnes maze. Testing
	36	Barnes maze. Long-term memory retention
	40	T-maze
	44	Forced swim test
**Group**	**Day**	**Test**
3	1	Social preference
	5	Female intruder test
	9	Male intruder test
	13	Two intruders test
	17	Habituation and startle reflex

Group	Day	Test
4	1	Social preference
	5	Female intruder test
	9	Male intruder test
	13	Two intruders test
	17	Habituation and startle reflex
**Group**	**Day**	**Test**
5	1	Sucrose preference test. Chip adaptation
	2	Sucrose preference test. Chip adaptation
	3	Sucrose preference test. Chip adaptation
	4	Sucrose preference test. Adaptation to IntelliCage
	5	Sucrose preference test. Adaptation to IntelliCage
	6	Sucrose preference test. Adaptation to IntelliCage
	7	Sucrose preference test. Adaptation to IntelliCage
	8	Sucrose preference test. Adaptation to IntelliCage
	9	Sucrose preference test. Adaptation to IntelliCage
	10	Sucrose preference test. Adaptation to IntelliCage
	11	Sucrose preference test. Sucrose preference
	12	Sucrose preference test. Sucrose preference
	13	Sucrose preference test. Sucrose preference
	14	Sucrose preference test. Sucrose preference
	15	Sucrose preference test. Sucrose preference
**Group**	**Day**	**Test**
6	1	Behavior in home cage
	2	Behavior in home cage
	3	Behavior in home cage

Supplementary Table 2 (Table S2)

List of primers used in the paper		
**Target**	**Primer name**	**Primer sequence 5' -> 3'**
Akkermansia muciniphila	AMUC- F	CAGCACGTGAAGGTGGGGAC
	AMUC-R	CCTTGCGGTTGGCTTCAGAT
16S rRNA	16S-F	TCCTACGGGAGGCAGCAG
	16S-R	ATTACCGCGGCTGCTGG
28S rRNA	28S-F	CCTGGCGCTAAACCATTCGT
	28S-R	AAAGCCCGCAGAGACAAACC
E. coli	guaB-F	TGCTTTCCGCAGCAATGGAT
	guaB-R	CTGCGGATCAGTCACCACAC
Mouse *β-tubulin (Tubb5)*	betaTub F	TGAAGCCACAGGTGGCAAGTAT
	betaTub R	CCAGACTGACCGAAAACGAAGT
Mouse Nos2	Nos2_F	CAGGGTCACAACTTTACAGGGA
	Nos2_R	CACTTCTGCTCCAAATCCAACG
Mouse Il-1β	Il1b_F	TGAAGTTGACGGACCCCAAA
	Il1b_R	TGATGTGCTGCTGCGAGATT
Mouse Tnf-α	Tnfa_F	CCCTCACACTCAGATCATCTTCT
	Tnfa_R	GGCACCACTAGTTGGTTGTCTTT
Mouse Cox2	Cox2-F	CCAGCACTTCACCCATCAGT
	Cox2-R	ACCCAGGTCCTCGCTTATGA

Supplementary Table 3 (Table S3)

Blood metabolic profiles as revealed by nuclear magnetic resonance (NMR) spectroscopy

Metabolite		*Muc2-/- vs.* C57Bl/6				*Muc2+/+ vs*. C57Bl/6			*Muc2-/- vs. Muc2+/+*		
	**Test**	**p-value**	**Test**	**Z**	**p-value**	**Test**	**Z**	**p-value**	**Test**	**Z**	**p-value**
2-hydroxyisovalerate	Kruskal-Wallis	0,0372	Mann–Whitney *u*-test	2,418973	0,015565	Mann–Whitney *u*-test	1,436265	0,150928	Mann–Whitney *u*-test	-1,36067	0,173618
Ketoleucine	Kruskal-Wallis	0,00001	Mann–Whitney *u*-test	3,74185	0,000183	Mann–Whitney *u*-test	2,45677	0,014020	Mann–Whitney *u*-test	-3,43948	0,000583
Leucine	Kruskal-Wallis	0,0235	Mann–Whitney *u*-test	2,30558	0,021135	Mann–Whitney *u*-test	2,30558	0,021135	Mann–Whitney *u*-test	0,34017	0,733730
Isobutyrate	Kruskal-Wallis	0,0162	Mann–Whitney *u*-test	1,70084	0,088974	Mann–Whitney *u*-test	2,45677	0,014020	Mann–Whitney *u*-test	1,77643	0,075663
2-ketoisovalerate	Kruskal-Wallis	0,0017	Mann–Whitney *u*-test	3,06151	0,002202	Mann–Whitney *u*-test	0,34017	0,733730	Mann–Whitney *u*-test	-2,98592	0,002827
Lactate	Kruskal-Wallis	0,0027	Mann–Whitney *u*-test	-0,11339	0,909722	Mann–Whitney *u*-test	-2,98592	0,002827	Mann–Whitney *u*-test	-2,83473	0,004587
Acetone	Kruskal-Wallis	0,0015	Mann–Whitney *u*-test	1,77643	0,212295	Mann–Whitney *u*-test	3,59066	0,037636	Mann–Whitney *u*-test	1,62525	0,241322
Pyruvate	Kruskal-Wallis	0,0126	Mann–Whitney *u*-test	2,00321	0,045155	Mann–Whitney *u*-test	2,53236	0,011330	Mann–Whitney *u*-test	1,54965	0,121225
Acetylcarnitine	Kruskal-Wallis	0,0131	Mann–Whitney *u*-test	-1,02050	0,307490	Mann–Whitney *u*-test	-2,75914	0,005796	Mann–Whitney *u*-test	-1,92762	0,053903
Choline	Kruskal-Wallis	0,00001	Mann–Whitney *u*-test	-3,36388	0,000769	Mann–Whitney *u*-test	-3,74185	0,000183	Mann–Whitney *u*-test	-3,06151	0,002202
Carnitine	Kruskal-Wallis	0,0237	Mann–Whitney *u*-test	-2,45677	0,014020	Mann–Whitney *u*-test	-2,15440	0,031210	Mann–Whitney *u*-test	0,11339	0,909722
Betaine	Kruskal-Wallis	0,0049	Mann–Whitney *u*-test	-2,98592	0,002827	Mann–Whitney *u*-test	-2,00321	0,045155	Mann–Whitney *u*-test	1,54965	0,121225
Glucose	Kruskal-Wallis	0,0471	Mann–Whitney *u*-test	2,07880	0,037636	Mann–Whitney *u*-test	2,07880	0,037636	Mann–Whitney *u*-test	-0,26458	0,791337
Fumarate	Kruskal-Wallis	0,0087	Mann–Whitney *u*-test	1,85203	0,064023	Mann–Whitney *u*-test	-1,47406	0,140466	Mann–Whitney *u*-test	-2,83473	0,004587
Histidine	Kruskal-Wallis	0,0006	Mann–Whitney *u*-test	2,45677	0,014020	Mann–Whitney *u*-test	3,28829	0,001008	Mann–Whitney *u*-test	2,30558	0,021135
Phenylalanine	Kruskal-Wallis	0,0424	Mann–Whitney *u*-test	2,07880	0,037636	Mann–Whitney *u*-test	2,15440	0,031210	Mann–Whitney *u*-test	0,18898	0,850107

Supplementary Table 4 (Table S4)

Brain metabolic profiles as revealed by nuclear magnetic resonance (NMR) spectroscopy

**Metabolite**		***Muc2-/- vs.* C57Bl/6**				***Muc2+/+ vs.* C57Bl/6**			***Muc2-/- vs.* Muc2+/+**		
	**Test**	**p-value**	**Test**	**Z**	**p-value**	**Test**	**Z**	**p-value**	**Test**	**Z**	**p-value**
Myo-inositol	Kruskal-Wallis	0,028	Mann–Whitney *u* -test	-2,08167	0,037374	Mann–Whitney *u* -test	-2,19089	0,028460	Mann–Whitney *u* -test	1,27802	0,201244
Scillo-inositol	Kruskal-Wallis	0,0221	Mann–Whitney *u* -test	-2,56205	0,010406	Mann–Whitney *u* -test	-2,00832	0,044611	Mann–Whitney *u* -test	-0,54772	0,583883
Glycine	Kruskal-Wallis	0,0154	Mann–Whitney *u* -test	-2,56205	0,010406	Mann–Whitney *u* -test	-2,00832	0,044611	Mann–Whitney *u* -test	-1,27802	0,201244
Inosinate	Kruskal-Wallis	0,0122	Mann–Whitney *u* -test	1,44115	0,149542	Mann–Whitney *u* -test	2,55604	0,010588	Mann–Whitney *u* -test	-2,19089	0,028460
